# Major facility superfamily sugar transporter protein *SsMFSST1* regulates *Sporisorium scitamineum* mating, pathogenicity, and sugar transport/absorption

**DOI:** 10.1128/spectrum.01956-24

**Published:** 2024-12-31

**Authors:** Yi Zhang, Yongding He, Meixin Yan, Shilong Zhang, Sisi Zhou, Wankuan Shen

**Affiliations:** 1 College of Agriculture, South China Agricultural University, Guangzhou, China; 2 Sugarcane Research Laboratory, South China Agricultural University 12526 , Guangzhou, China; 3 Sugarcane Research Institute, Guangxi Academy of Agricultural Sciences, Nanning, China; The Hebrew University-Hadassah School of Dental Medicine, Jerusalem, Israel

**Keywords:** sugarcane, *Sporisorium scitamineum*, Major Facilitator Superfamily (MFS) sugar transporter, sexual mating, pathogenicity

## Abstract

Sugarcane smut caused by *Sporisorium scitamineum* is a global sugarcane disease, and studying its molecular pathogenesis is crucial for discovering new prevention and control targets. This study was based on the transcriptome sequencing data of two isolates with different pathogenicities (*Ss16* and *Ss47*) of the *S. scitamineum* and screened out a gene encoding the Major Facility Superfamily (MFS) sugar transporter protein and named it *SsMFSST1*. Knockout mutants (*∆SsMFSST1^+^* and *∆SsMFSST1^−^*) and complementary mutants (*COMMFSST1^+^* and *COMMFSST1^−^*) were obtained through polyethylene glycol (PEG)-mediated protoplast transformation technology. On this basis, the function of gene *SsMFSST1* was analyzed. The research results showed that the sexual mating ability of the knockout mutants significantly decreased compared with the wild type, while the sexual mating ability of the complementary mutants was almost restored to the level of the wild type. After the addition of exogenous small molecular signaling substance cyclic adenosine monophosphate (cAMP) or tryptophol required for the sexual mating of *S. scitamineum*, the sexual mating ability of the knockout mutants was almost restored to the level of the wild type. It was observed that the expression levels of the key genes *Uac1* for cAMP synthesis and *Aro8* for tryptophol synthesis were significantly lower in the knockout mutants compared with the wild type. However, the expression levels of these genes in the complementary mutants were restored to the wild-type levels. The pathogenicity testing also found a significant decrease in the pathogenicity of combinations containing mutants. On YePSA medium, the gene *SsMFSST1* does not affect the growth, spore morphology, colony morphology, and oxidative stress ability of the haploid spores of *S. scitamineum*. Therefore, we speculate that the *SsMFSST1* gene may regulate the expression of key genes related to the synthesis pathway of the small molecule signaling substance cAMP or tryptophol, affecting the synthesis of the signaling substance cAMP or tryptophol, thereby affecting the sexual mating and pathogenicity of *S.scitamineum*. In addition, this gene also regulates the transport/absorption of fructose and lactose in *S. scitamineum*. In summary, this study identified a novel pathogenic gene in the *S. scitamineum*—the MFS sugar transporter gene, providing a molecular basis for understanding the pathogenic mechanism of the *S. scitamineum*. On the other hand, it also enriches the biological functions of this gene in fungi.

**IMPORTANCE:**

Sugarcane is an important economic crop, but the sugarcane smut disease caused by *S. scitamineum* severely damages the yield and quality of sugarcane, causing huge economic losses on the sugarcane industry. Therefore, it is very necessary to study the pathogenic mechanism of *S. scitamineum*, especially at the molecular level. This manuscript identified a new pathogenic gene and discovered a new pathogenic mechanism of this gene in *S. scitamineum*, enriched the molecular pathogenesis of *S. scitamineum*, and provided a new target for the prevention and control of sugarcane smut disease.

## INTRODUCTION

Sugarcane (*Saccharum officinarum*) belongs to the C4 crop category and is a significant sugar-producing crop in China. China ranks as the world’s third-largest producer of sugarcane sugar. In recent years, China’s sugarcane planting area reaches 1.3 million hectares per year, the total sugarcane output reaches 90 million tons per year, and the production of sugarcane sugar is 10 million tons per year. Sugarcane smut disease caused by *Sporisorium scitamineum* was first discovered in Natal, South Africa, in 1877 and has since spread to sugarcane cultivation areas worldwide, becoming a globally important sugarcane disease (1, 2). Sugarcane smut, also known as sugarcane whip smut, affects the apical buds of young shoots during the early growth and development of sugarcane (3). Infected plants exhibit symptoms, such as elongated and pale green leaves, and slender and branching stems. The most distinctive feature is the formation of a downward-curled dark smut on the shoot tip of the diseased sugarcane. The smut gradually forms 2–4 months after the plant is infected and reaches its maximum length after 6–7 months (4, 5). The central core of the smut whip is composed of host tissues, surrounded by a thick layer of sporidia. Different parts of the whip have different distributions of spores, with mature spores at the top, more mature spores in the middle, and some immature spores at the base (6). The causative agent of sugarcane smut disease belongs to the subdivision basidiomycotina fungi, which is a typical dimorphic fungus. It exhibits two opposite mating types designated as “+” and “−”. Haploid sporidia reproduce through budding and reproduce into yeast-like colonies, which are not pathogenic (7). Only haploid sporidia with two opposite mating types undergo sexual mating to produce pathogenic dikaryotic hyphae, which then infect plant tissues (8–10). Therefore, the sexual mating of haploid sporidia of two opposite mating types to form dikaryotic hyphae is a crucial aspect of the pathogenicity of *S. scitamineum* (11).

In *S. scitamineum*, the formation of pathogenic dikaryotic hyphae through sexual mating of two opposite haploid sporidia is crucial for the development of pathogenicity. Therefore, understanding the sexual mating of the *S. scitamineum* is a prerequisite for investigating its pathogenicity. In recent years, with the advancement of biotechnology, there has been some progress in unraveling the molecular mechanisms underlying the sexual mating and pathogenicity of the *S. scitamineum*. For instance, the cAMP/PKA signaling pathway can promote the sexual mating of haploid sporidia of two opposite mating types to form dikaryotic hyphae by increasing ROS levels in *S. scitamineum*, facilitating their invasion of the host (12). Cai et al. (13) knocked out the gene *SsCI80130* encoding squalene monooxygenase in *S. scitamineum*, the growth rate of the knockout mutant was significantly reduced, and the sexual mating ability was significantly reduced. Further studies have shown that *SsCI80130* affects the sexual mating and pathogenicity of *S. scitamineum* by regulating the synthesis of small molecule signaling substances cyclic adenosine monophosphate (cAMP) and tryptophol, which are necessary for sexual mating. Cai et al. (14) studied a conserved histidine kinase gene *SsSln1* of *S. scitamineum* and found that when *SsSln1* was deleted, the sexual mating ability and pathogenicity of *S.scitamineum* were enhanced. Further studies showed that *SsSln1* and cAMP/PKA jointly regulate the transcription of pheromone response transcription factor (*SsPRF1*) gene, thereby regulating the sexual mating and pathogenicity of *S. scitamineum*. Some studies have also found that the gene encoding the pheromone response factor (*Prf1*) in *S. scitamineum* is involved in sexual mating and pathogenicity, indicating that this gene plays an important role in pheromone signaling and filamentation of *S. scitamineum* (15).

Sugar transport proteins are widely present in all living organisms, including microbes, plants, and animals. Major Facilitator Superfamily (MFS) sugar transport proteins is one of the largest and most diverse superfamilies of transport proteins. These MFS transport proteins typically consist of 400–600 amino acids, exhibiting high similarity in their primary structure and usually containing 12 transmembrane domains (16–18). Sugar as a crucial carbon source not only provides energy for organisms but also participates in various intracellular metabolic activities. At present, some reports have identified a small number of MFS sugar transporter genes from different fungi, and it has been found that knocking out or silencing MFS sugar transporter genes in some fungi affects the growth and development of fungi and weakens their pathogenicity. For instance, in *Verticillium dahliae*, the two MFS sugar transporter genes *VdST3* and *VdST12* reduce pathogenicity to cotton by inhibiting the growth of fungal hyphae and colonies (19). In *Ustilago maydis*, the absence of MFS sugar transporter *Srt1* does not affect the growth of the pathogen on the culture medium but significantly reduces the pathogenicity of *U. maydis*. Infected corn plants have only chlorotic lesions or produce small “tumor-like” spore masses (20). Furthermore, Liu et al. (21) identified an MFS sugar transporter *CgMFS1* and found that *CgMFS1* not only plays a role in sugar transport but also plays a key role in antioxidant stress and pathogenicity in *Colletotrichum gloeosporioides*. In addition, similar findings have also been made in bacteria. Joko et al. (22) identified an MFS sugar transporter gene *mfsX* in *Dickeya dadantii* and found that knockout mutant successfully reduced pathogenicity of *D. dadantii* to Chinese cabbage, potato, and chicory, and also found that the flagellum number, swimming, and swarm movement rate were significantly reduced in the mutant compared with the wild type, and the formation of biofilm was seriously reduced. It can be seen that MFS sugar transporter plays an important role in the formation of fungal and bacterial pathogenicity. However, there is currently no reported information on the functions of MFS sugar transporter in *S. scitamineum*. Therefore, it is very necessary to explore the function of MFS sugar transporter in *S. scitamineum*, especially its role in sexual mating and pathogenicity.

Based on the transcriptome sequencing data of different pathogenic strains *Ss16* and *Ss47* previously isolated in our laboratory (23), we identified the significantly upregulated gene *SsMFSST1* in the strongly pathogenic strain *Ss16*. The predicted protein encoded by this gene is the MFS sugar transporter. In this study, we aimed to investigate the biological function of the *SsMFSST1* gene in *S. scitamineum* and elucidate the molecular basis of its pathogenic mechanism. We employed polyethylene glycol (PEG)-mediated protoplast transformation to generate knockout and complementary mutants for the *SsMFSST1* gene. Phenotypic analysis and pathogenicity assessments were conducted on the wild-type, knockout mutants, and complementary mutants.

## MATERIALS AND METHODS

### Characterization of the *SsMFSST1* gene sequence

Based on the previous transcriptome sequencing data from our laboratory, the gene *SsMFSST1*, encoding MFS sugar transport protein, showed significant (*P ≤ 0.05*) differential expression between the strongly pathogenic strain *Ss16* and the weakly pathogenic strain *Ss47* (23) . The protein encoded by the *SsMFSST1* gene was analyzed using the pI/MW tool to determine its isoelectric point and molecular weight (https://web.expasy.org/compute_pi). Amino acid sequence analysis was performed using the NCBI database, and the conservative domains of the protein encoded by the *SsMFSST1* gene were obtained through Blast comparisons (https://blast.ncbi.nlm.nih.gov/Blast.cgi). Additionally, the DNA sequence was translated into an amino acid sequence using an online tool (https://www.novopro.cn/tools/translate.html). The subcellular localization of the protein encoded by the *SsMFSST1* gene was predicted using the online tool ProtComp (http://linux1.softberry.com/). Prediction of the secondary structure of the protein encoded by the *SsMFSST1* gene using PRABI-Doua (https://doua.prabi.fr/software/cap3). The three-dimensional structure of the protein was predicted using SWISS-MODEL (https://swissmodel.expasy.org/). Systematic phylogenetic analysis of the protein encoded by the *SsMFSST1* gene was conducted in MEGA 7, employing the neighbor-joining method (24, 25).

### Strains and growth conditions

#### Experimental material

The wild-type haploid strains *Ss16^+^* and *Ss16^−^* were isolated and identified in our laboratory, and they are stored in a −80°C freezer (4). The culture media used in this study include YePS liquid medium (yeast extract 1%, peptone 2%, and sucrose 2%), YePS soft medium (yeast extract 1%, peptone 2%, sucrose 2%, and agar 0.7%), YePSA solid medium (yeast extract 1%, peptone 2%, sucrose 2%, and agar 2%), YePSS medium (yeast extract 1%, peptone 2%, sucrose 2%, D-sorbitol 18.17%, and agar 2%), and MM medium (K_2_HPO_4_ 0.205%, KH_2_PO_4_ 0.145%, NH_4_NO_3_ 0.05%, (NH_4_)_2_SO_4_ 0.03%, FeSO_4_ 0.001%, CaCl_2_ 0.001%, Glucose 0.2%, Z-Buffer 50%, pH 7.0).

#### Growth test

The wild-type haploid sporidial (*Ss16^+^*, *Ss16^−^*), knockout mutants (*∆SsMFSST1^+^*, *∆SsMFSST1^−^*), and complementary mutants (*COMMFSST1^+^*, *COMMFSST1^−^*) were cultured in 15 mL of YePS liquid medium at 28°C with shaking at 200 rpm for 24 h. Subsequently, sporidia were diluted in fresh YePS liquid medium to achieve a cell density of 10^5^ cells/mL. The cultures were then incubated under the same conditions for an additional 48 h. At intervals of 6 h, cell growth of the wild-type haploid sporidial, knockout mutants, and complementary mutants was monitored by measuring the optical density at 600 nm (OD_600_) using a spectrophotometer (NanoDrop 2000C). For the sexual mating experiment, equal volumes of opposite mating-type wild-type haploid sporidial, knockout mutants, or complementary mutants were mixed and cultured on YePSA medium (control group) and YePSA medium supplemented with 5 mM cAMP or 0.02 mM tryptophol. The cultures were incubated at 28°C under dark conditions for 42 h, and photographs were taken for documentation. For abiotic stress tolerance experiments, 100 µg/mL Congo red (CR), 50 µg/mL sodium dodecyl sulfate (SDS), or 500 mM NaCl was added to YePSA medium and MM medium for evaluation. The cultures were then incubated at 28°C under dark conditions for 48 h, and photographs were taken for documentation.

### Nucleic acid manipulation

Genomic DNA was extracted using the modified CTAB method (26). PCR amplification was performed using Phanta Max Super-Fidelity DNA Polymerase (Vazyme, P505). DNA fragments were purified using the FastPure Gel DNA Extraction Mini Kit (Vazyme, DC301). Total RNA was extracted using TRIZOL (Vazyme, R401), and cDNA was synthesized using HiScript III RT SuperMix (Vazyme, R323). The concentration and purity of nucleic acids were measured using NanoDrop ND-1000 (Thermo Fischer Scientific, Wilmington, DE, USA).

### Construction of *SsMFSST1* gene knockout and complementary mutants

The knockout strategy for the *SsMFSST1* gene involves homologous recombination using PEG-mediated protoplast transformation with double-stranded DNA fragments (27). Amplification of two flanking fragments of the *SsMFSST1* gene using genomic DNA. Amplification of overlapping upstream and downstream fragments using hygromycin resistance gene (hygromycin phosphotransferase, *Hpt*) (27). Then, the two flanking fragments were fused to the upstream and downstream fragments of the *Hpt* gene. These two fusion fragments were used for subsequent protoplast transformation to obtain knockout mutants. Figure S1 shows a schematic diagram of gene knockout.

The complementation of the *SsMFSST1* gene also employed PEG-mediated protoplast transformation (12). The difference is that the complementary mutants not only replace the *Hpt* gene with the target gene in the knockout mutants but also carry the selectable *Zeocin* resistance gene (Zeocin, also known as phleomycin D1, was isolated from a mutant strain of *Streptomyces verticillus*). Figure S2 shows a schematic diagram of gene complementation. The primer design was based on the NCBI *S. scitamineum* genome sequence LK056689.1. Table 1 lists all the primers related to the construction and validation of the knockout mutants and complementary mutants.

**TABLE 1:** Primers used in this study

Name	Primer sequences (5’−3’)	Description
SsMFSST1-LB-F	GGATTAAGCGCTCGTTGCTG	Deletion construction
SsMFSST1-LB-R	GTCGTGACTGGGAAAACCCTGTTCGGTGGTCGGGTGAAG	
SsMFSST1-RB-F	GGTCATAGCTGTTTCCTGTGTGATTTCGATCGGGGTGCTGAG	
SsMFSST1-RB-R	GGTCAAACTCGCACTTTCGG	
Hpt-LB-F	CAGGGTTTTCCCAGTCACGAC	
Hpt-LB-R	GGTCAAGACCAATGCGGAGC	
Hpt-RB-F	GCAAGACCTGCCTGAAACCG	
Hpt-RB-R	TCACACAGGAAACAGCTATGACC	
SsMFSST1-IN-F	CCAAACAAACGGCATGCAGA	PCR verification
SsMFSST1-IN-R	CGGCGCAGAACATTACATGG	
SsMFSST1-OU-F	CGAGTCGAGTTGGACAACCA	
SsMFSST1-OU-R	GCGTTCCGACATGGCTTTAC	
MFSST1COM-F	ATCCAAGCTCAAGCTAAGCTTGACGTGGTTGTCGACTGACT	Complementation construction
MFSST1COM-R	CAGCAAGATCTAATCAAGCTTCGCAACTTCCACAGGCTAGC	
COM-HPT-LB-F	GCGCGCGTAATACGACTCAC	
Zeocin-R	GAAGTGCACGCAGTTGCCG	
Situ-F	CTCCGTGTTGATGCTGGGAC	
COM-HPT-RB-R	CGAGCATTCACTAGGCAACCA	
Zeocin-IN-F	CTGTGATCAGCAGCCAAT	PCR verification
Zeocin-IN-R	GTCAACTTGGCCATGGTG	
SsMFSST1-qF	GTTGAAACCCGACAACAGCC	qRT-PCR
SsMFSST1-qR	AGAGCAGGGAGAAGACGGAT	
Aro8-qF	CCTGGTGTTGCGTTCATTCC	
Aro8-qR	CAAGCTCGGGCATCGTCTTA	
Uac1-qF	CTGACGGAGATGTAGCCAAAG	
Uac1-qR	AACGAGACAAGGAGGGAGTA	
Actin-qF	ACAGGACGGCCTGGATAG	
Actin-qR	TCACCAACTGGGACGACA	

### Analysis of gene expression

To evaluate the effect of *SsMFSST1* gene on the synthesis of the small molecule signaling substances cAMP and tryptophol required for sexual mating of *S. scitamineum*, we used quantitative real-time (qRT)-PCR to determine the expression levels of the key gene *Uac1* (a key gene in the cAMP synthesis pathway, encoding adenylyl cyclase) and the key gene *Aro8* (a key gene in the synthesis pathway of tryptophol, encoding tryptophol synthase) (28). The experiment involved three sexual mating combinations: the wild-type combination (*Ss16^+^ + Ss16^−^*), the knockout mutant combination (*∆SsMFSST1^+^ + ∆SsMFSST1^−^*), and the complementary mutant combination (*COMMFSST1^+^ + COMMFSST1^−^*). For each combination, cultures were incubated at 28°C for 48 h, and total RNA was extracted, and cDNA was synthesized every 12 h during this period. qRT-PCR was performed using the ChamQTM Universal SYBR qPCR Master Mix (Vazyme, Q711) to detect the expression levels of the *Uac1* and *Aro8* genes. The *ACTIN* gene was used as the internal reference, and the 2^−ΔΔCt^ method was employed to calculate the relative expression levels (29). Three biological repeats each containing three technical replicates for each sample were performed.

Using the same method as above, we measured the expression of gene *SsMFSST1* under three culture conditions (haploid sporidial growth, sexual mating, and sugarcane bud infection process). The expression levels of the *SsMFSST1* gene were monitored every 12 h within a 72 h period. Three biological repeats each containing three technical replicates for each sample were performed. The primer sequences used are provided in Table 1.

### Assay of sugar utilization ability in *SsMFSST1* gene knockout and complement mutants

The wild-type haploid sporidial (*Ss16^+^*, *Ss16^−^*), knockout mutants (*∆SsMFSST1^+^*, *∆SsMFSST1^−^*), and complementary mutants (*COMMFSST1^+^*, *COMMFSST1^−^*) were cultured in 10 mL of YePS liquid medium at 28°C with shaking at 200 rpm for 24 h. Subsequently, sporidia were diluted in MM liquid medium (with 2% glucose, 2% fructose, 2% lactose, and 2% maltose as the sole carbon source) to achieve a cell density of 10^5^ cells/mL. The cultures were then incubated under the same conditions for a period of time. At 6 h intervals, cell growth of the wild-type haploid sporidial, knockout mutants, and complementary mutants was monitored by measuring the optical density at 600 nm (OD_600_) using a spectrophotometer (NanoDrop 2000C).

### Sugar content determination

The wild-type haploid sporidial (*Ss16^+^*, *Ss16^−^*), knockout mutants (*∆SsMFSST1^+^*, *∆SsMFSST1^−^*), and complementary mutants (*COMMFSST1^+^*, *COMMFSST1^−^*) were cultured in 10 mL of YePS liquid medium at 28°C with shaking at 200 rpm for a period of time. Then, an appropriate amount of fresh spore solution was transferred to an MM liquid medium containing 10 mL of fructose and lactose as the only carbon source. The initial OD_600_ value was adjusted to 0.01 and incubated in the shaker for 48 h. The content of fructose and lactose in the solution was measured every 12 h using the Fructose Content Detection Kit (JL-T0895, Shanghai, China) and Lactose Content Detection Kit (JL-T1071, Shanghai, China), respectively.

### Assay of the pathogenicity of the *SsMFSST1* gene knockout mutants and complementary mutants

The wild-type (*Ss16^+^*, *Ss16^−^*), knockout mutants (*∆SsMFSST1^+^*, *∆SsMFSST1^−^*), and complementary mutants (*COMMFSST1^+^*, *COMMFSST1^−^*) were inoculated into 10 mL of YePS liquid medium and cultured at 28°C with constant shaking at 200 rpm for 24 h. Subsequently, the sporidia were collected by centrifugation and washed twice with ddH_2_O, resuspended in fresh YePS liquid medium, and finally adjusted the concentration to 2 × 10^9^ sporidia /mL. Equal volumes of sporidial liquids of opposite mating types were mixed, and then 200 µL each was pipetted and injected into the stem growth point of the susceptible sugarcane variety “ROC22” (4–5 young leaves stage). A total of seven combinations, including (*Ss16^+^ + Ss16^−^*), (*Ss16^+^ + COMMFSST1^−^*), (*Ss16^−^ +COMMFSST1^+^*), (*COMMFSST1^+^ + COMMFSST1^−^*), (*∆SsMFSST1^+^ + ∆SsMFSST1^−^*), (*Ss16^+^ + ∆SsMFSST1^−^*), and (*Ss16^−^ + ∆SsMFSST1^+^*), were inoculated with 16 sugarcane plants per combination (four plants per pot, 4 pots per combination). The wild-type combination (*Ss16^+^ + Ss16^−^*) served as the positive control, and sterile water inoculation was used as the negative control. The inoculated plants were placed in the greenhouse for 4 months, and the incidence of sugarcane smut was observed and recorded once a month; diseased plants were marked in each survey to avoid repeated surveys, and the black whips were covered with plastic bags to prevent the spread of sporidia. Finally, calculate the number of diseased plants and incidence rate.

### Microscopy

Images were taken using an Axio Observer Z1 microscope (Zeiss, Jena, Germany) equipped with a sCMOS camera (PCO Edge, Kelheim, Germany).

### Statistic analysis

Data were expressed as the means ± standard error (SE). Differences among different treatments were analyzed using IBM SPSS Statistics 20.

## RESULTS

### Identification and characterization of the *SsMFSST1* gene

**Fig 1:**
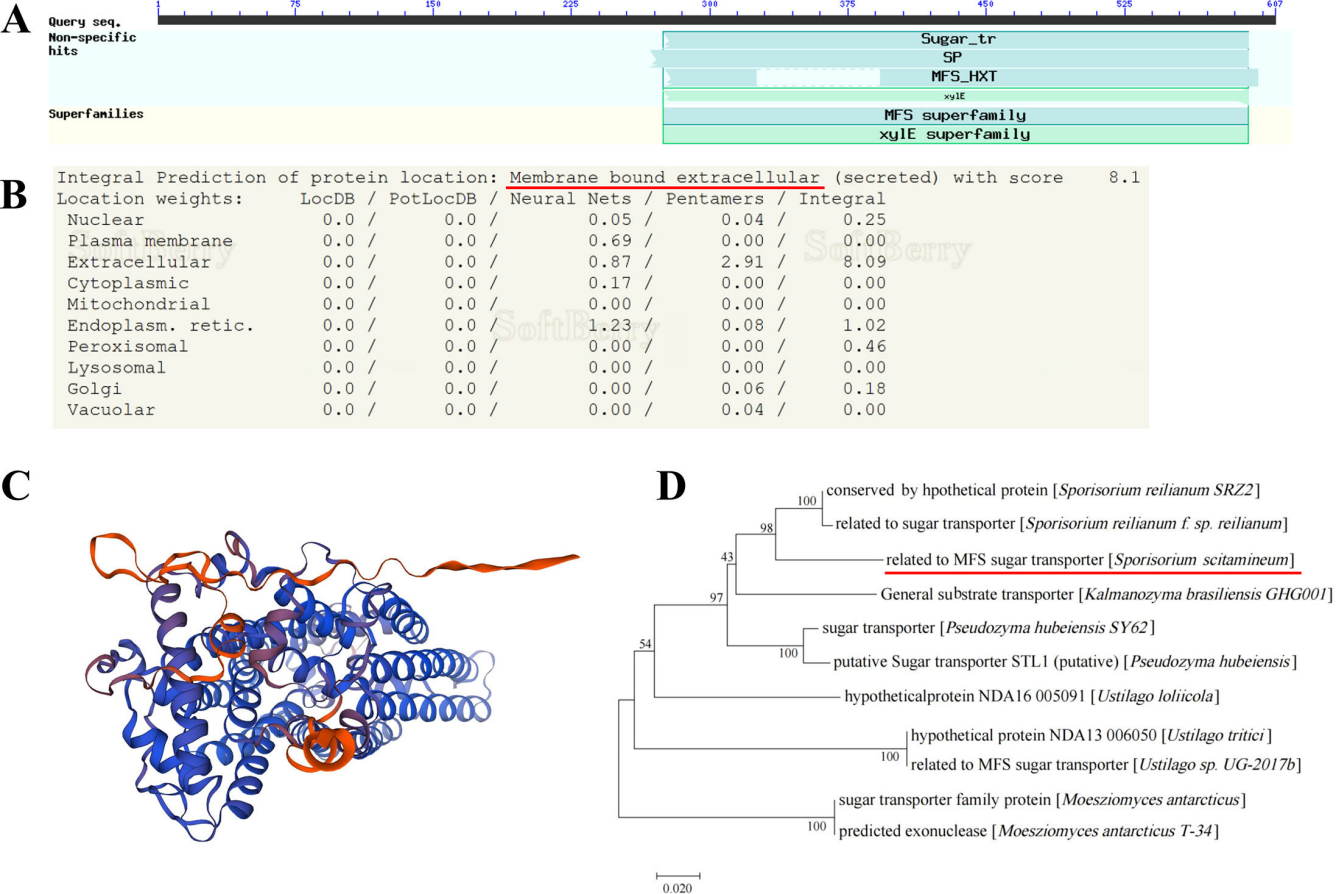
The structural domain and phylogenetic analysis of the *SsMFSST1* gene encoding the MFS sugar transporter in the *S. scitamineum*. (**A**) The predicted protein encoded by the *SsMFSST1* gene is the MFS sugar transporter. (**B**) Subcellular localization prediction of the protein encoded by the *SsMFSST1* gene. (**C**) The prediction of the tertiary structures of the protein encoded by the *SsMFSST1* gene. Different colors represent the consistency of the protein sequence. Blue indicates consistency greater than 80%, purple represents consistency between 70% and 80%, and yellow indicates consistency less than 50%. (**D**) Phylogenetic tree analysis of the protein encoded by the *SsMFSST1* gene, with Arabic numerals on the tree nodes indicating the confidence level of the phylogenetic tree (using the bootstrap method with 1,000 replicates). The protein is highlighted with a red horizontal line.

The *SsMFSST1* gene encoding the MFS sugar transporter protein was identified as a significantly differentially expressed gene (*P ≤ 0.05*) in the *S. scitamineum* strains *Ss16* (strong pathogenicity) and *Ss47* (weak pathogenicity) (23). The full length of this gene is 1,839 bp. Protein sequence alignment on NCBI revealed that the protein encoded by *SsMFSST1* (NCBI: protein accession no. CDR88814.1) is the MFS sugar transporter protein consisting of 607 amino acids. The predicted isoelectric point of the protein is 9.88, and the molecular weight is 69.2 kDa (Fig. 1A). The subcellular localization prediction indicates the association with membrane-bound organelles (Fig. 1B). The predicted secondary structure of the protein reveals the following proportions: α-helix, 42.34%; extended strand, 17.30%; β-turn, 4.78%; and random coil, 35.58%. *SsMFSST1* gene-predicted 3D structure is highly similar (0.76 coverage) to the gene *A0A4U7KVZ3_9BASI* of Major Facilitator Superfamily (MFS) profile domain-containing protein in *Sporisorium graminicola* (Fig. 1C). SsMFSST1 was predicted to have nine transmembrane helices (Fig. S3). Phylogenetic analysis demonstrates high homology of this protein with *Sporisorium reilianum SRZ2* conserved monocarboxylate permease and *Sporisorium reilianum f.* sp*. reilianum* conserved monocarboxylate permease, suggesting that the SsMFSST1 is highly conserved in *Sporisorium* (Fig. 1D). Meanwhile, we also found that this protein is highly conserved with MFS transporters reported with role in pathogenesis in other fungi, such as *Verticillium dahliae*, *Puccinia striiformis f. sp. tritici*, *Colletotrichum siamense*, and *Mycosarcoma maydis* (Fig. S4).

### Molecular construction of *SsMFSST1* knockout mutants and complementary mutants

**Fig 2:**
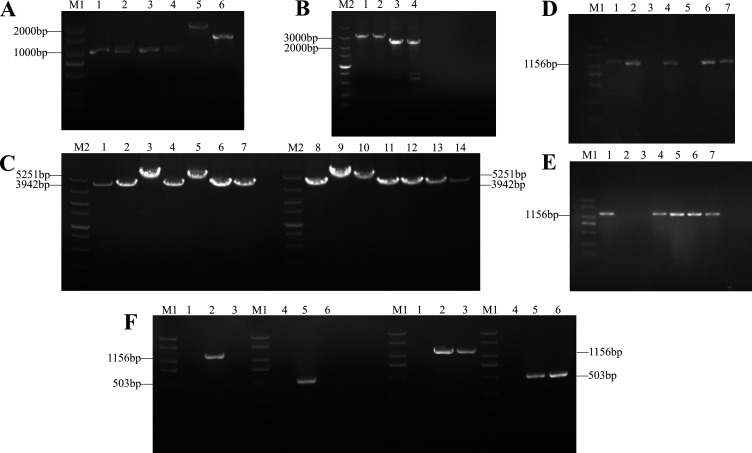
Construction and verification of *SsMFSST1* gene knockout mutants and complementary mutants. M1 represents the 2,000 marker, M2 represents the 5,000 marker. (**A**) PCR amplification. Lanes 1, 3, and 2, 4 represent the left and right flanking fragments of the *SsMFSST1* gene in the wild type (*Ss16^+^* and *Ss16^−^*). Lanes 5 and 6 represent the upstream and downstream fragments of the *Hpt* gene, respectively. (**B**) Fusion PCR. Lanes 1 and 2 represent the fused fragments of the left flanking fragment and upstream fragment in two wild-type backgrounds, respectively. Lanes 3 and 4 represent the fused fragments of the right flanking fragment and downstream fragment, respectively. (**C**) PCR external verification of the knockout mutants *∆SsMFSST1^+^* (left) and *∆SsMFSST1^−^* (right) in the *Ss16^+^* and *Ss16^−^* backgrounds. Lanes 3, 5, 9, and 10 represent the knockout mutants, and the remaining lanes represent the wild type. (**D**) PCR internal verification of the knockout mutant *∆SsMFSST1^+^* in the *Ss16^+^* backgrounds. Lanes 3 and 5 represent the knockout mutant, and the remaining lanes represent the wild type. (**E**) PCR internal verification of the knockout mutant *∆SsMFSST1^−^* in the *Ss16^−^* background. Lanes 2 and 3 represent the knockout mutant, and the remaining lanes represent the wild type. (**F**) PCR verification of the complementary mutants (*COMMFSST1*^+^ and *COMMFSST1^−^*). The left half of Figure F shows the PCR verification for *COMMFSST1^+^*, lanes (1, 4) and (3, 6) represent the knockout mutant *∆SsMFSST1^+^*. Lanes (2, 5) represent the complementary mutant in the *∆SsMFSST1^+^* background. Similarly, the right half of Figure F represents the complementary mutant *COMMFSST1^−^* in the *∆SsMFSST1^−^* background with lanes (2, 5) and (3, 6), and lanes (1, 4) represent the knockout mutant *∆SsMFSST1^−^*.

The construction methods of *SsMFSST1* knockout mutants and complementary mutants are described in the Materials and Methods section. Using wild-type genomic DNA as a template, *SsMFSST1*-LB-F/R and *SsMFSST1*-RB-F/R were used as primers to amplify two flanking fragments with lengths of 927 and 1,003 bp respectively. In the same way, the *Hpt* gene was used as a template, and Hpt-LB-F/R and Hpt-RB-F/R are primers that amplify upstream and downstream fragment, with lengths of approximately 2 and 1.5 kb respectively (Fig. 2A). Subsequently, *SsMFSST1*-LB-F/Hpt-LB-R and Hpt-RB-F/*SsMFSST1*-RB-R were used as primer pairs to fuse the two flanking fragments with the upstream and downstream fragments of the *Hpt* gene, respectively. The length of the fusion fragments is approximately 3 and 2.5 kb respectively (Fig. 2B). These two fused fragments were used for the subsequent wild-type protoplast transformation. As we expected, we successfully obtained knockout mutants (*∆SsMFSST1^+^* and *∆SsMFSST1^−^*). We used the external primer pair *SsMFSST1*-OU-F/R to detect a 5.251 bp band in the knockout mutants, but only a 3,942 bp band in the wild type (Fig. 2C). The internal primer pair *SsMFSST1*-IN-F/R was able to detect a 1,156 bp band in the wild type, but no band was detected in the knockout mutants (Fig. 2D and E). Similarly, after the complementary mutants (*COMMFSST1^+^* and *COMMFSST1^−^*) was successfully obtained, the internal primer pair *SsMFSST1*-IN-F/R could detect a 1156 bp band, and the primer pair *Zeoncin*-IN-F/R could detect a 503 bp band, while no band was detected in the knockout mutants (Fig. 2F).

### Effect of *SsMFSST1* gene on haploid sporidial morphology, colony morphology, and growth rate of *S. scitamineum*

**Fig 3:**
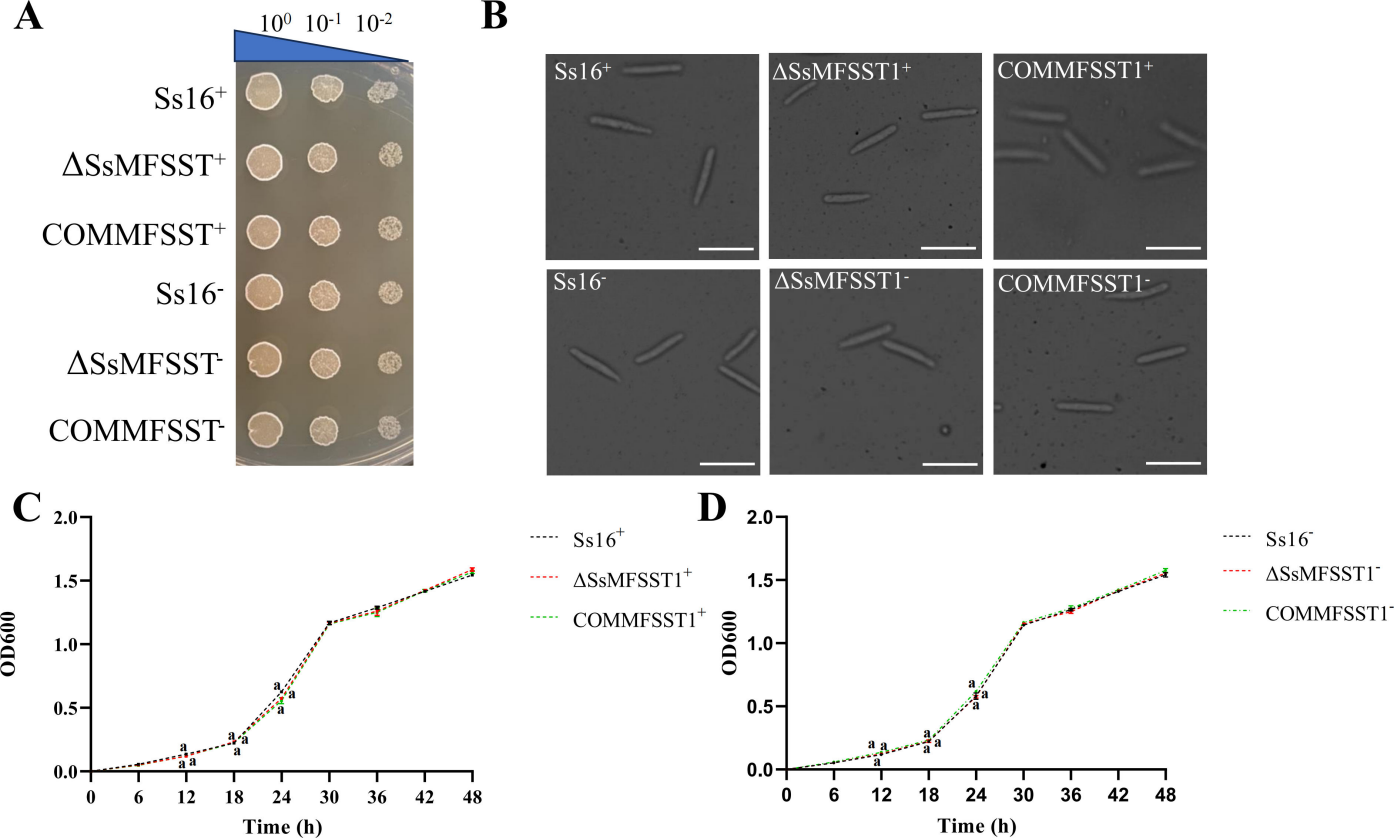
Impact of the *SsMFSST1* gene on the haploid phenotype of *S. scitamineum*. (**A**) Colony morphology of the wild type (*Ss16^+^*, *Ss16^−^*), knockout mutants (*∆SsMFSST1^+^*, *∆SsMFSST1^−^*) and complementary mutants (*COMMFSST1^+^*, *COMMFSST1^−^*) after 48 h of incubation at 28°C on YePSA solid medium. (**B**) Microscopic images of haploid sporidia from the wild-type, knockout mutants, and complementary mutants (observed under a 40× microscope after 48 h of cultivation in YePS liquid medium at 28°C), where the white bar represents 25 µm. (**C-D**) Growth curves of haploid sporidia. Haploid sporidia of the wild-type, knockout mutants, and complementary mutants were inoculated to YePS liquid medium and cultured for 48 h. Each sample contains three replicates. Bars represent standard errors. Same lowercase letters denote no differences at a 0.05 significance level.

The results showed that the haploid sporidial and colony morphology of the knockout mutants (*∆SsMFSST1^+^*, *∆SsMFSST1^−^*) were the same as those of the wild-type (*Ss16^+^*, *Ss16^−^*) and the complementary mutants (*COMMFSST1^+^*, *COMMFSST1^−^*). This indicates that the *SsMFSST1* gene does not affect the haploid sporidial and colony morphology of *S. scitamineum* (Fig. 3A and B) . It was found that the growth rate of the knockout mutants was roughly the same as that of the wild type, indicating that the *SsMFSST1* gene did not affect the growth rate of *S.scitamineum* (Fig. 3C and D).

### Effect of *SsMFSST1* gene on abiotic stress tolerance

**Fig 4:**
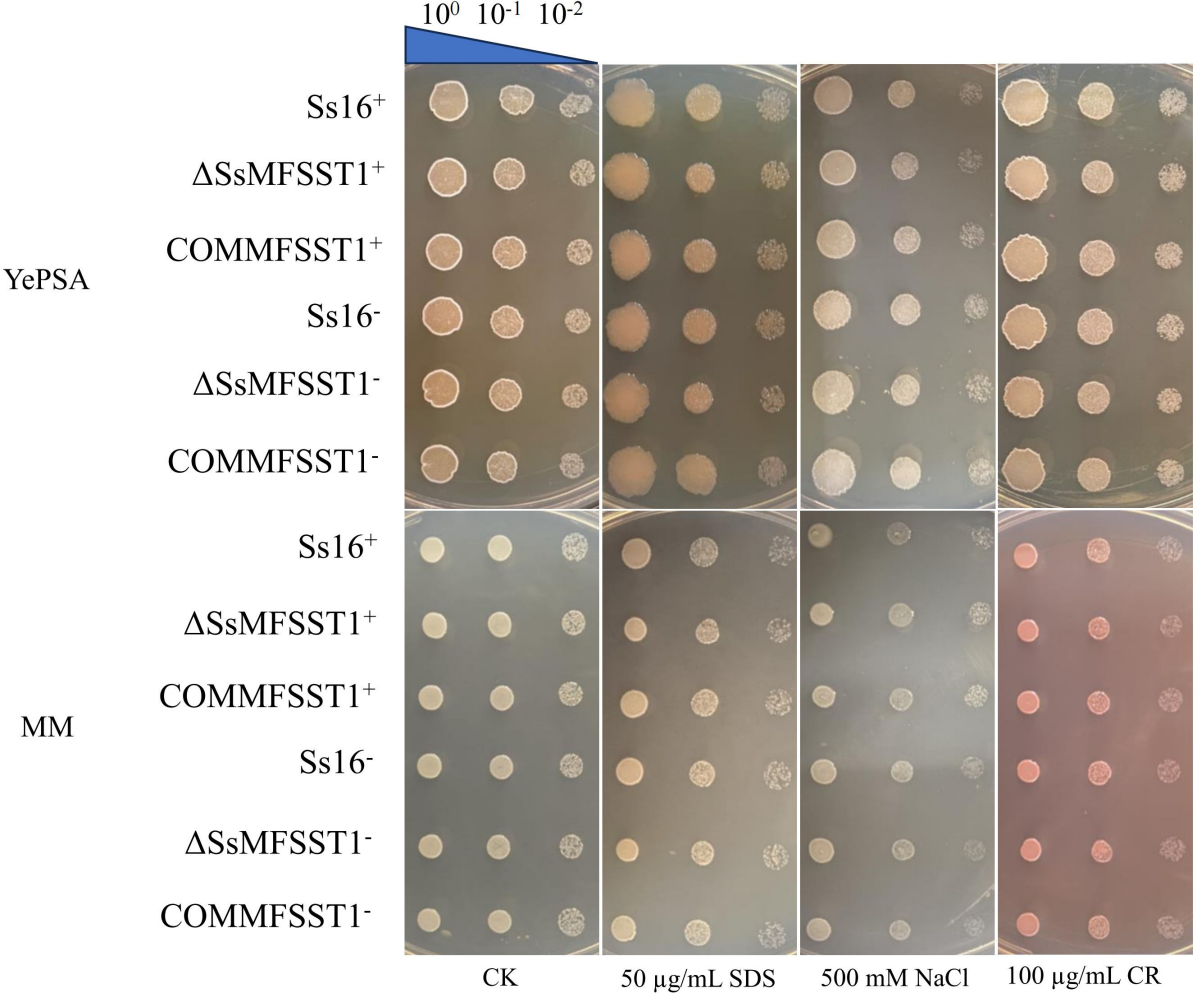
The influence of the *SsMFSST1* gene on abiotic stress in *S. scitamineum*. Wild-type (*Ss16^+^*, *Ss16^−^*), knockout mutants (*∆SsMFSST1^+^*, *∆SsMFSST1^−^*), and complementary mutants (*COMMFSST1^+^*, *COMMFSST1^−^*) were spotted on YePSA and MM media supplemented with CR (100 µg/mL), SDS (50 µg/mL), and NaCl (500 mM). Samples were cultured at 28°C for 48 h. YePSA and MM media without additional substances were used as controls. Using YePSA as the culture medium, the image of the control group CK is the same as the image of A in Fig. 3. It has been reused in this figure for ease of comparison.

We assessed the tolerance of wild type (*Ss16^+^*, *Ss16^−^*), knockout mutants (*∆SsMFSST1^+^*, *∆SsMFSST1^−^*), and complementary mutants (*COMMFSST1^+^*, *COMMFSST1^−^*) by adding abiotic stresses, such as cell wall stress (SDS or CR) and osmotic stress (NaCl). Upon the addition of SDS, CR, or NaCl, we observed that the growth rate of the knockout mutants was almost no different from that of the wild-type and complementary mutants. This suggests that the *SsMFSST1* gene is not associated with the stress response related to cell wall stress or osmotic stress (Fig. 4).

### Determination of gene expression level of *SsMFSST1*

**Fig 5:**
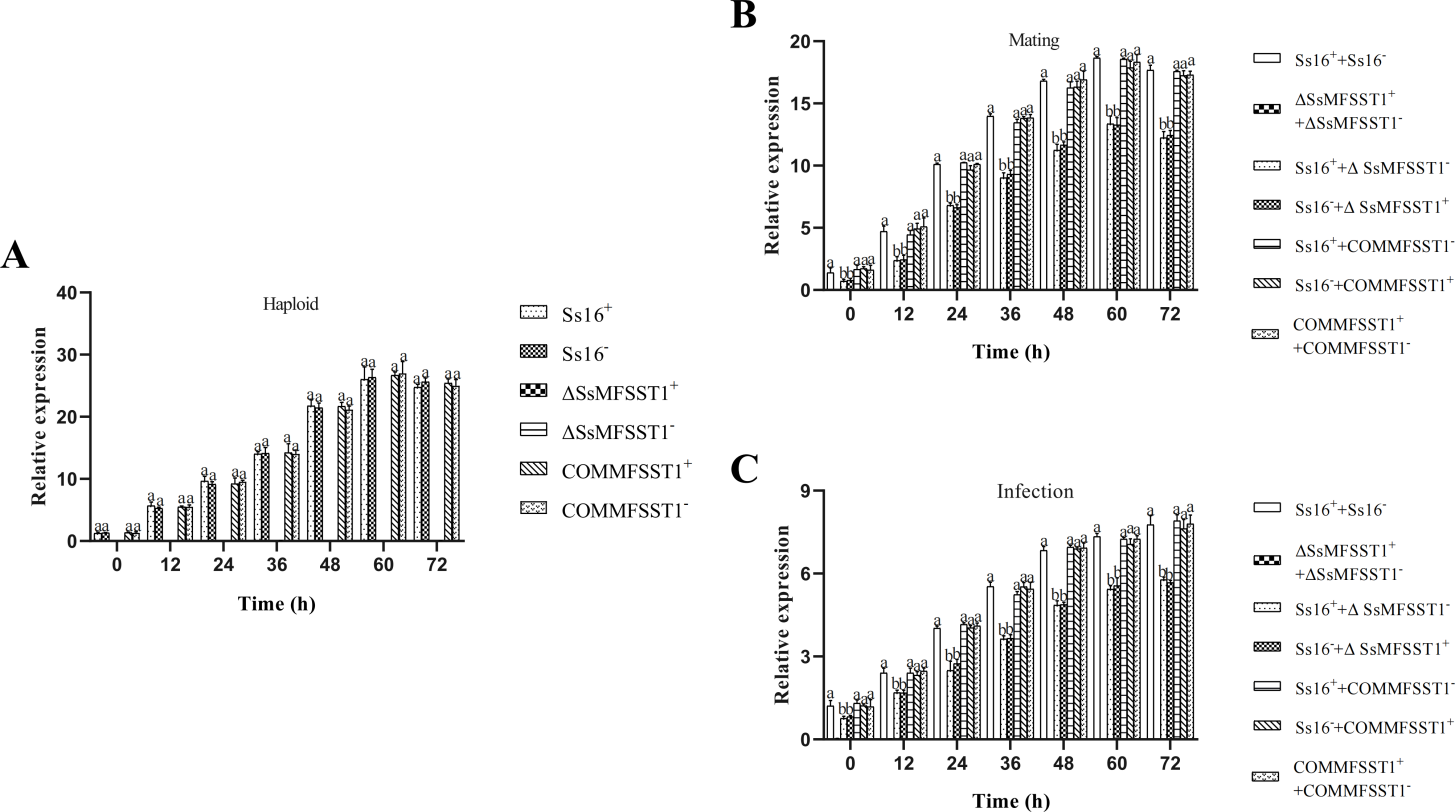
Expression levels of the *SsMFSST1* gene at different stages. (**A**) Expression levels during haploid sporidial growth. (**B**) Expression levels during sexual mating culture. (**C**) Expression levels during sugarcane bud infection process. Error bars represent standard errors. Different lowercase letters indicate significant differences at the 0.05 level.

During the haploid sporidial growth, the gene expression levels of the wild-type (*Ss16^+^*, *Ss16^−^*) and complementary mutants (*COMMFSST1^+^*, *COMMFSST1^−^*) continuously increased over time, reaching a peak at 60 h and eventually leveling off. However, in the knockout mutants (*∆SsMFSST1^+^*, *∆SsMFSST1^−^*), no expression of the *SsMFSST1* gene was detected (Fig. 5A). In the sexual mating culture, the gene expression levels in the wild-type and knockout mutant combinations (*Ss16^+^ + ∆SsMFSST1^−^* and *Ss16^−^ + ∆SsMFSST1^+^*) were significantly lower than that in the wild-type combination (*Ss16^+^ + Ss16^−^*) and the combination containing complementary mutants (*COMMFSST1^+^ + COMMFSST1^−^*, *Ss16^+^ + COMMFSST1^−^*, *Ss16^−^ + COMMFSST1^+^*). Notably, the expression of the *SsMFSST1* gene was not detected in the knockout mutant combination (*ΔSsMFSST1^+^ + ΔSsMFSST1^−^*) (Fig. 5B). During the sugarcane bud infection process, the expression level of the *SsMFSST1* gene followed almost the same trend as observed during sexual mating culture (Fig. 5C).

### Effect of *SsMFSST1* gene on sexual mating compatibility of *S.scitamineum*

**Fig 6:**
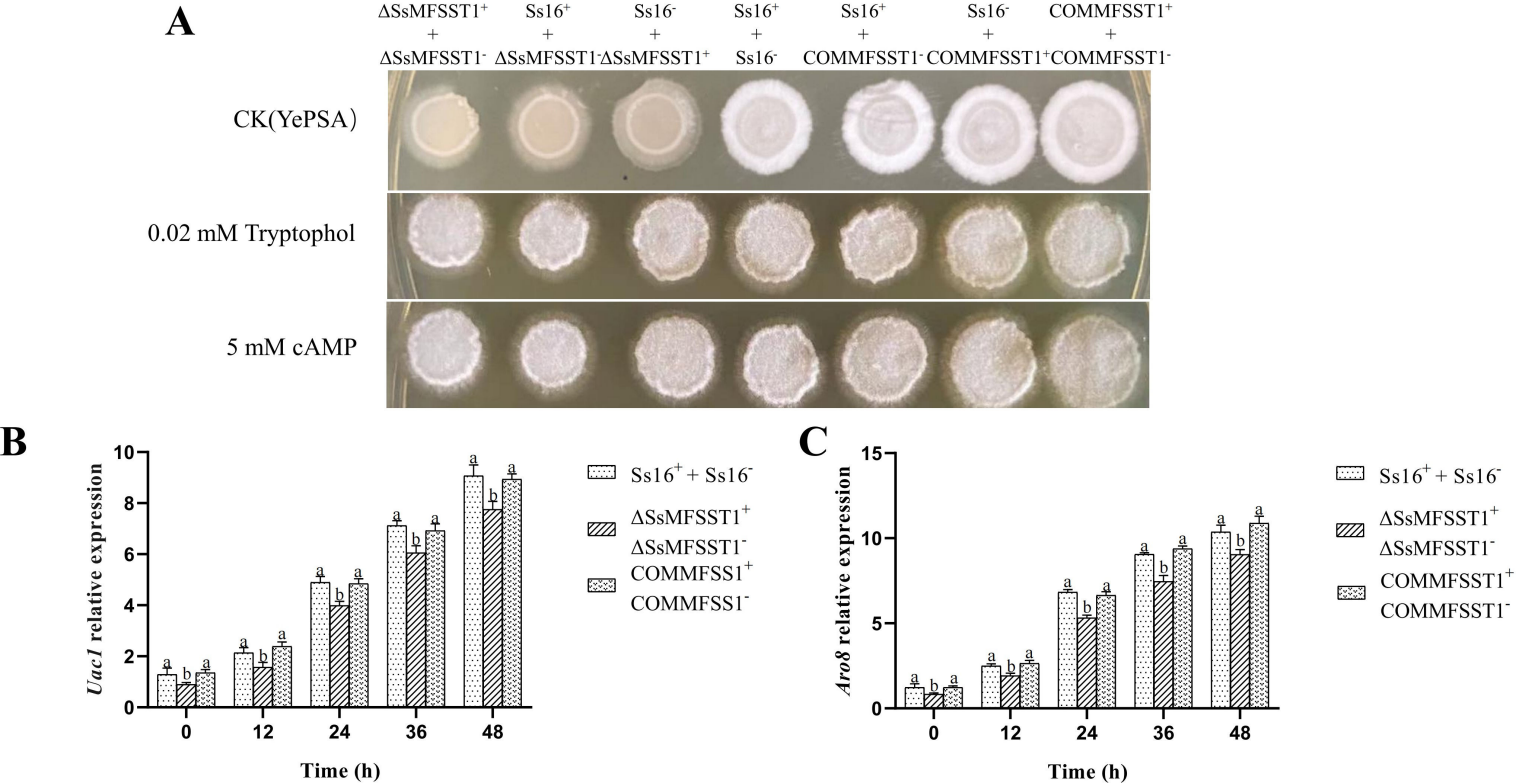
The impact of the *SsMFSST1* gene on the sexual mating of *S.scitamineum*. (**A**) Influence of adding cAMP (5 mM) or tryptophol (0.02 mM) on the sexual mating ability on YePSA medium. Photographs were taken 42 h after inoculation, with untreated YePSA medium as a control. (**B-C**) Expression levels of the *Uac1* and *Aro8* gene. Error bars represent standard errors. Different lowercase letters indicate significant differences at the 0.05 level.

On YePSA medium, it was found that the wild-type combination (*Ss16^+^ + Ss16^−^*) and the combination containing complementary mutants (*COMMFSST1^+^ + COMMFSST1^−^*, *Ss16^+^ + COMMFSST1^−^* and *Ss16^−^ + COMMFSST1^+^*) produced a large number of white villous hyphae, indicating that wild-type and complementary mutants are capable of normal sexual mating. However, the knockout mutant combination (*∆SsMFSST1^+^ + ∆SsMFSST1^−^*) and the combination containing knockout mutants (*Ss16^+^ + ∆SsMFSST1^−^* and S*s16^−^ + ∆SsMFSST1^+^*) produced less white villous hyphae, indicating that the sexual mating ability of the knockout mutants was reduced (Fig. 6A). After adding exogenous small molecule signaling substances cAMP or tryptophol, which are necessary for sexual mating of *S. scitamineum* on the YePSA medium, the knockout mutant combination (*∆SsMFSST1^+^ + ∆SsMFSST1^−^*) and the combination containing knockout mutants (*Ss16^+^ + ∆SsMFSST1^−^* and S*s16^−^ + ∆SsMFSST1^+^*) produced a large number of white villous hyphae (Fig. 6A). This indicates that the *SsMFSST1* gene affects the synthesis of small molecule signaling substances cAMP and tryptophol required for sexual mating of *S. scitamineum*. To further validate this result, we measured the expression levels of key genes involved in cAMP and tryptophol synthesis, in the 48 h period of sexual mating, the expression levels of the cAMP synthesis key gene *Uac1* increase with the extension of cultivation time. Notably, the gene expression levels of the *Uac1* in the knockout mutant combination (*∆SsMFSST1^+^ + ∆SsMFSST1^−^*) are significantly lower compared with the wild-type combination (*Ss16^+^ + Ss16^−^*), and the complementary mutant combination (*COMMFSST1^+^ + COMMFSST1^−^*) returned to the wild-type level (Fig. 6B). The expression levels of the key gene *Aro8* involved in tryptophol synthesis show a similar trend to *Uac1* (Fig. 6C). Observations of fungal morphology under a 40× microscope reveal no difference in hyphal growth among the knockout mutant combination (*∆SsMFSST1^+^ + ∆SsMFSST1^−^*), wild-type combination (*Ss16^+^ + Ss16^−^*), wild-type knockout mutant combinations (*Ss16^+^ + ∆SsMFSST1^−^* and *Ss16^−^ + ∆SsMFSST1^+^*), and complementary mutant combination (*COMMFSST1^+^ + COMMFSST1^−^*) (Fig. S5).

### Effect of *SsMFSST1* gene on the sugar utilization ability of *S. scitamineum*

**Fig 7:**
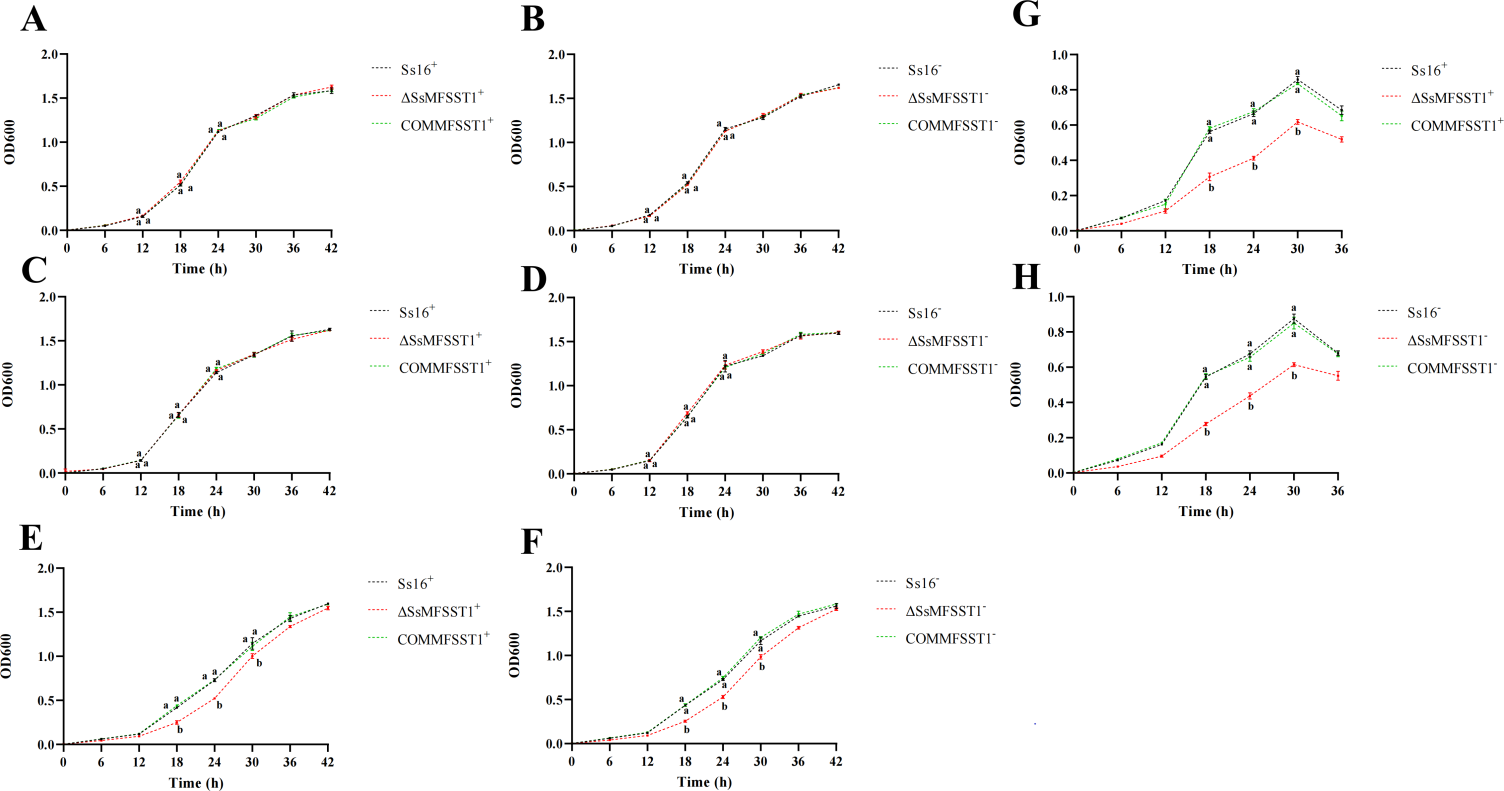
Effect of *SsMFSST1* gene on the sugar utilization ability of *S. scitamineum*. (**A-B**) Growth curve of knockout mutants with glucose as the sole carbon source added to MM liquid culture medium. (**C-D**) Growth curve of knockout mutants with maltose as the sole carbon source added to MM liquid culture medium. (**E-F**) Growth curve of knockout mutants with fructose as the sole carbon source added to MM liquid culture medium. (**G-H**) Growth curve of knockout mutants with lactose as the sole carbon source added to MM liquid culture medium. Error bars represent standard errors. Different lowercase letters indicate significant differences at the 0.05 level.

To identify the effect of *SsMFSST1* gene on sugar utilization ability in MM liquid culture medium with glucose, fructose, lactose, and maltose as the sole carbon sources. The research results found that the growth rate of knockout mutants (*∆SsMFSST1^+^*, *∆SsMFSST1^−^*) in MM liquid culture medium with glucose and maltose as the sole carbon sources was roughly the same as that of wild-type (*Ss16^+^*, *Ss16^−^*) and complementary mutants (*COMMFSST1^+^*, *COMMFSST1^−^*) (Fig. 7A through D). In MM liquid culture medium with fructose or lactose as the sole carbon sources, it was found that the growth rate of knockout mutants was significantly reduced (Fig. 7E through H). Further observation was conducted on the growth of wild-type, knockout mutants, and complementary mutants in MM solid culture medium with fructose or lactose as the sole carbon sources. The research results also found that the growth colonies of knockout mutants were significantly smaller than those of wild-type and complementary mutants (Fig. S6), indicating the *SsMFSST1* gene may be closely related to the utilization of fructose and lactose.

### *SsMFSST1* gene is related to the transport/absorption of fructose and lactose

**Fig 8:**
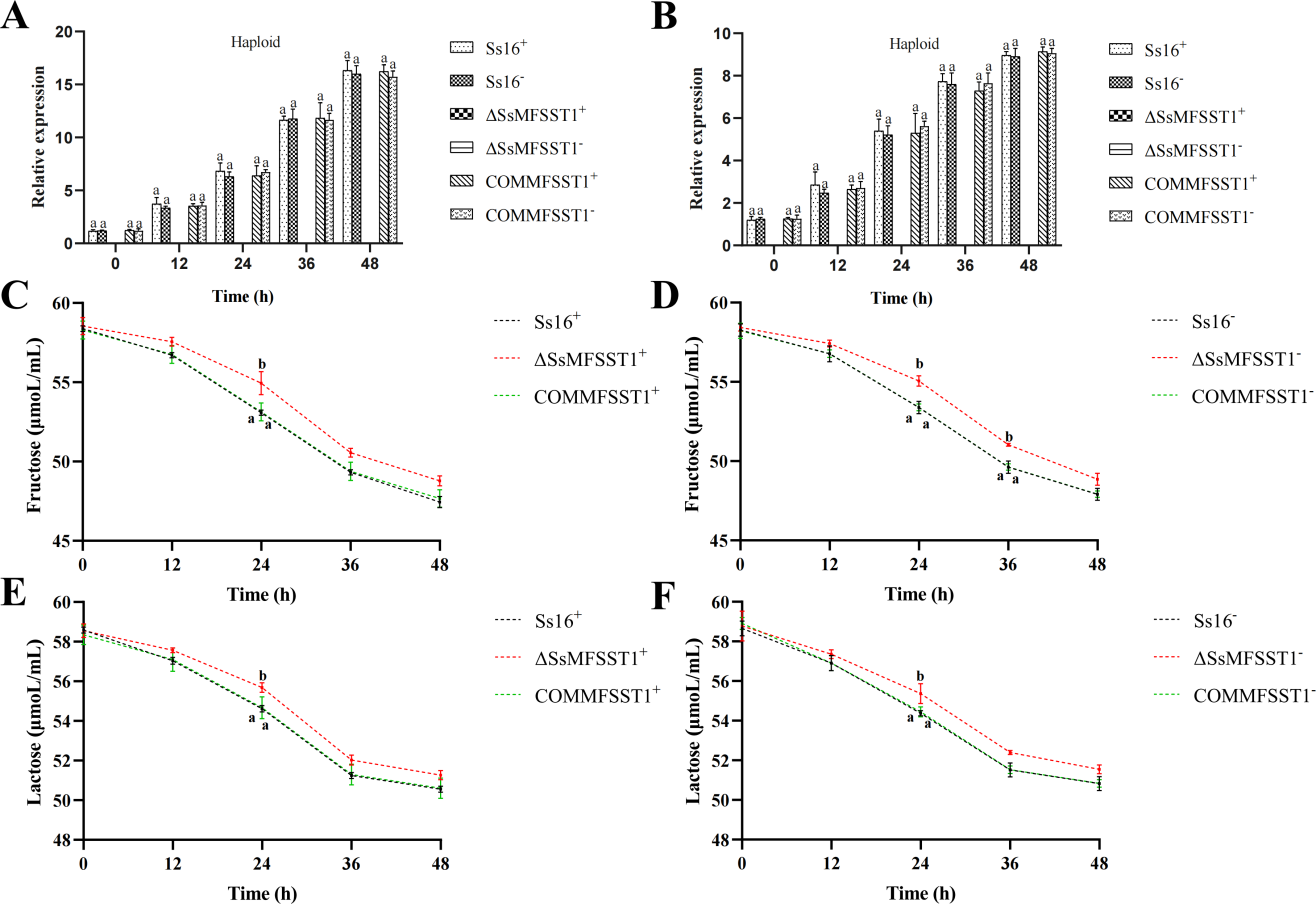
*SsMFSST1* gene is related to the absorption of fructose and lactose. (**A**) The expression levels of *SsMFSST1* gene were measured in MM liquid culture media with fructose as the sole carbon sources. (**B**) The expression levels of *SsMFSST1* gene were measured in MM liquid culture media with lactose as the sole carbon sources. (**C-F**) The content of fructose and lactose in MM liquid culture medium with fructose and lactose as the sole carbon sources. Error bars represent standard errors. Different lowercase letters indicate significant differences at the 0.05 level.

Knockout mutants were significantly inhibited in MM culture medium with fructose or lactose as the sole carbon sources. To further validate this result, within 48 h, the expression level of *SsMFSST1* gene and the content of fructose or lactose in the haploid sporidial culture medium with fructose or lactose as the sole carbon sources were simultaneously detected. The results showed that not only was the expression of the gene not detected in the knockout mutant (Fig. 8A and B), but the content of fructose or lactose in the knockout mutant medium was significantly or not significantly higher than that in the wild-type and complementary mutant (Fig. 8C through F). This indicates that the MFS sugar transporter protein encoded by the *SsMFSST1* gene of *S. scitamineum* may be closely related to the transport/absorption of fructose or lactose.

### Effect of *SsMFSST1* gene on pathogenicity of *S.scitamineum*

**Fig 9:**
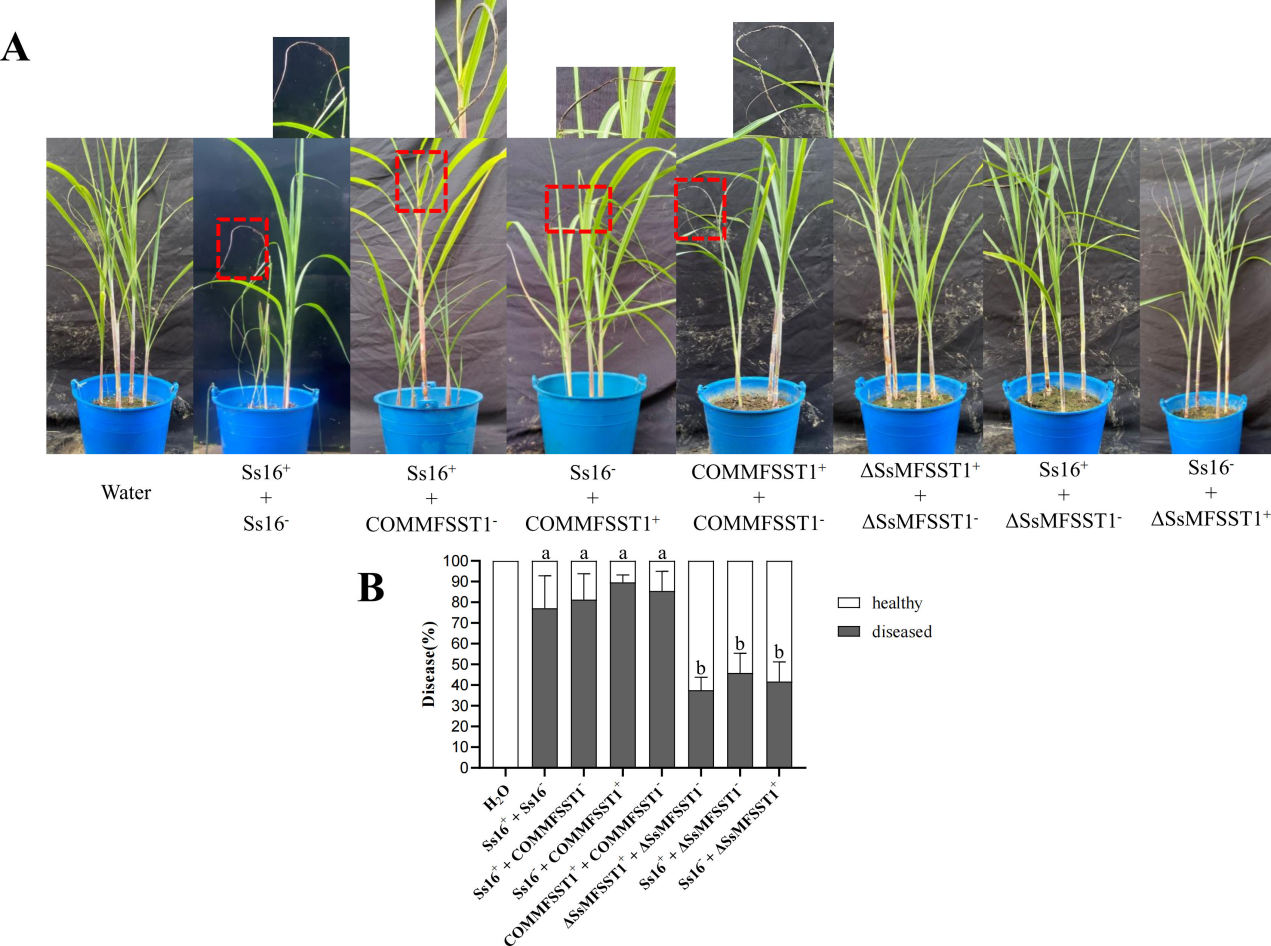
Effect of *SsMFSST1* gene in the pathogenicity of *S. scitamineum*. (**A**) Symptoms of sugarcane smut. Black whips are marked with red dashed boxes. (**B**) Incidence rate of different treatment combinations. Error bars represent standard errors. Different lowercase letters indicate significant differences at the 0.05 level.

To identify the effect of *SsMFSST1* gene on the pathogenicity of *S. scitamineum*, we inoculated mixed sporidial suspension of opposite mating types to the susceptible variety “ROC22” of sugarcane smut disease. A total of seven combinations were inoculated, including (*Ss16^+^ + Ss16^−^*), (*Ss16^+^ + COMMFSST1^−^*), (*Ss16^−^ +COMMFSST1^+^*), (*COMMFSST1^+^ + COMMFSST1^−^*), (*∆SsMFSST1^+^ + ∆SsMFSST1^−^*), (*Ss16^+^ + ∆SsMFSST1^−^*), and (*Ss16^−^ + ∆SsMFSST1^+^*). The wild-type combination (*Ss16^+^ + Ss16^−^*) served as a positive control, and sterile water inoculation was the negative control (Fig. 9A). Inoculation with mixed sporidial suspensions lacking the knockout mutant combinations (*Ss16^+^ + Ss16^−^*, *Ss16^+^ + COMMFSST1^−^*, *Ss16^−^ +COMMFSST1^+^*, *COMMFSST1^+^ + COMMFSST1^−^*) resulted in high incidence rates, approximately 77%, 81%, 90%, and 85%, respectively. In contrast, inoculation with mixed sporidial suspensions containing the knockout mutant combinations (*∆SsMFSST1^+^ + ∆SsMFSST1^−^*, *Ss16^+^ + ∆SsMFSST1^−^*, *Ss16^−^ + ∆SsMFSST1^+^*) led to a significant reduction in disease incidence, with rates of 38%, 45%, and 42%, respectively (Fig. 9B). These results indicate that the *SsMFSST1* gene is involved in regulating the pathogenicity of *S.scitamineum*.

## DISCUSSION

Based on the transcriptome sequencing results of the strongly pathogenic strain *Ss16* and the weakly pathogenic strain *Ss47* of *S. scitamineum* (23), this study identified a encoding MFS sugar transporter gene *SsMFSST1*, among the significantly differentially expressed genes. The knockout mutants (*∆SsMFSST1^+^*, *∆SsMFSST1^−^*) and complementary mutants (*COMMFSST1^+^*, *COMMFSST1^−^*) were constructed using PEG-mediated protoplast transformation technology. Phylogenetic analysis revealed high conservation of *SsMFSST1* gene in *Sporisorium*. Additionally, protein subcellular localization prediction indicated association with membrane-bound extracellular. We predicted that this protein had nine transmembrane helices instead of the typical 12 transmembrane helices (18), which may vary among different software predictions and require further validation. It was also possible that this protein was not a typical MFS transporter protein. In this study, the expression of the *SsMFSST1* gene was not detected in the knockout mutants during the processes of haploid sporidial growth, sexual mating, and sugarcane bud infection process. Furthermore, the expression level of this gene in the complementary mutants was restored to the level as the wild type. These findings indicate that the knockout mutants and complementary mutants constructed in this study are of reliable quality and can be used for subsequent functional studies (30).

MFS sugar transport proteins play a crucial role in biological systems (31). Sugar substances serve as essential energy sources in living organisms, and the regulation of sugar transport proteins is vital for energy exchange between cells (32, 33). For fungi, sugar is the main source of energy in their life. Once the fungus has defects in the absorption of sugar substances, its cell metabolism and pathogenicity will be affected. Schuler et al. (34) identified a gene encoding a hexose transporter Hxt1 in *Ustilago maydis*, when *Hxt1* was deleted, knockout mutant significantly reduced the transport/absorption of hexose (glucose, fructose, or mannose), and the growth rate of knockout mutant was significantly reduced on culture media with hexose (glucose, fructose, or mannose) as the sole carbon source. Chang et al. (35) used host-induced silencing of a hexose transporter gene *PsHXT1* in *Puccinia striiformis f.sp. tritici. PsHXT1* gene silencing significantly inhibited the transport/absorption of hexose by knockout mutants during wheat infection, thereby affecting the normal growth and development of knockout mutants, resulting in a significant decrease in the number of spores and a significant decrease in pathogenicity. These reports indicate that MFS sugar transporters play an important role in the growth, development and pathogenesis of pathogenic fungi, mainly by regulating their sugar transport/absorption. Similar findings were also found in this study. We conducted an experiment on the sugar (glucose, fructose, lactose, and maltose) utilization ability of *SsMFSST1* gene. The results showed that the growth rate of knockout mutants was significantly reduced when they were cultured on MM medium with fructose or lactose as the only carbon source. Further research has found that this gene positively regulates the transport/absorption of fructose and lactose in *S. scitamineum*. This study only confirms that this gene affects the transport/absorption of fructose and lactose in *S. scitamineum*, but further in-depth research is needed to determine the extent of its impact on the transport/absorption of fructose and lactose.

The *S. scitamineum* is a typical dimorphic fungus, haploid sporidia have no pathogenicity, and only haploid sporidia of the two opposite mating types can produce white dikaryotic hyphae through sexual mating, and then they can be pathogenic. Therefore, sexual mating is the prerequisite for the pathogenicity of *S. scitamineum*. On the contrary, if sexual mating cannot be carried out normally, it will affect the formation of pathogenicity (36). It has been proven that the cAMP–PKA signaling pathway is closely related to the morphological transformation and pathogenicity of *S. scitamineum* (37, 38). Once cAMP synthesis is blocked, its sexual mating ability is affected, resulting in the inability to form dikaryotic hyphae and the inability to produce pathogenicity. However, after the exogenous addition of cAMP, a small molecule signaling substance required for sexual mating of *S. scitamineum*, its sexual mating ability was restored (12, 39). In addition, Wang et al. (40) found that the exogenous small molecule signal substance tryptophol has a significant impact on the sexual mating of *S.scitamineum*. For example, the AGC kinase gene *SsAgc1* knockout mutant in *S. scitamineum* has a weakened sexual mating ability, but after adding tryptophol, the sexual mating ability is restored. Therefore, cAMP or tryptophol is a small molecule signaling substance required for sexual mating of *S.scitamineum*. This study also found similar findings, the gene knockout mutants were tested by exogenously adding the small molecule signaling substance cAMP or tryptophol. The results showed that after exogenous addition of cAMP or tryptophol, the sexual mating ability of the knockout mutants was restored to the wild-type level, and a large number of white dikaryotic hyphae were produced. Microscopic observation showed that the restored hyphae were almost identical in shape to those produced by the wild type. More importantly, qRT-PCR detection results indicated a significant decrease in the expression levels of key gene *Aro8* related to tryptophol synthesis and key gene *Uac1* related to cAMP synthesis in the knockout mutants compared with the wild type, while the expression levels of the corresponding genes in the complementary mutants were restored to the wild-type level. These results indicate that *SsMFSST1* may regulate the synthesis of small molecule signaling substances cAMP or tryptophol required for sexual mating of *S. scitamineum* by regulating expression of key genes related to the cAMP or tryptophol synthesis pathway, and then regulating its sexual mating and pathogenicity (knockout mutations with exogenous cAMP or tryptophol can restore sexual mating ability, but whether pathogenicity can be restored still needs further verification). How does the *SsMFSST1*gene regulate the key genes involved in the cAMP and tryptophol synthesis pathway, and whether the sugar transported by this gene is involved in regulating the key genes involved in the cAMP and tryptophol synthesis pathway. These will require further in-depth research in the future.

### Conclusion

In summary, this study utilized PEG-mediated protoplast transformation technology to obtain knockout mutants and complementary mutants of the MFS sugar transporter gene *SsMFSST1* in *S. scitamineum*. Subsequently, comprehensive comparative analyses were conducted on the haploid sporidial morphology, colony hyphal morphology, growth rate, sexual mating ability, gene expression levels, abiotic stress tolerance, sugar utilization ability and field pathogenicity. The main research conclusions are as follows: (i) In the sexual mating experiment, the sexual mating ability of the knockout mutant was significantly reduced, and the pathogenicity experiment also found that the incidence rate of sugarcane inoculated with the knockout mutant was significantly reduced. This indicates that the gene is involved in the positive regulation of sexual mating and pathogenicity of *S. scitamineum* (ii). The *SsMFSST1* gene may regulate the expression of key genes in the synthesis pathway of small molecule signaling substances cAMP and tryptophol required for sexual mating of *S. scitamineum,* and then affects sexual mating and pathogenicity (iii). On YePSA medium, this gene does not affect the growth, spore morphology, colony morphology, and non-biological stress oxidative stress ability of the haploid spores of *S. scitamineum* (iv). The gene *SsMFSST1* regulates the transport/absorption of fructose and lactose of *S.scitamineum*, affecting sporidial growth (v). This study identified a new pathogenic gene (MFS sugar transporter gene) in *S. scitamineum.* This study enriches the molecular pathogenesis of *S. scitamineum* and provides a new target for the prevention and control of sugarcane smut disease.

## SUPPLEMENTAL MATERIAL

Supplemental material spectrum.01956-24-s0001.docx Fig. S1 to S6.

ASM does not own the copyrights to Supplemental Material that may be linked to, or accessed through, an article. The authors have granted ASM a non-exclusive, world-wide license to publish the Supplemental Material files. Please contact the corresponding author directly for reuse.
