# Supplementary material for: Major facility superfamily sugar transporter protein SsMFSST1 regulates Sporisorium scitamineum mating, pathogenicity, and sugar transport/absorption
Source: Microbiol Spectr. 2024 Dec 31;13(2):e01956-24. doi: 10.1128/spectrum.01956-24 (PMC11792463; doi:10.1128/spectrum.01956-24)
Supplement: Supplemental material — Fig. S1 to S6. [file spectrum.01956-24-s0001.docx]

**Supplementary materials
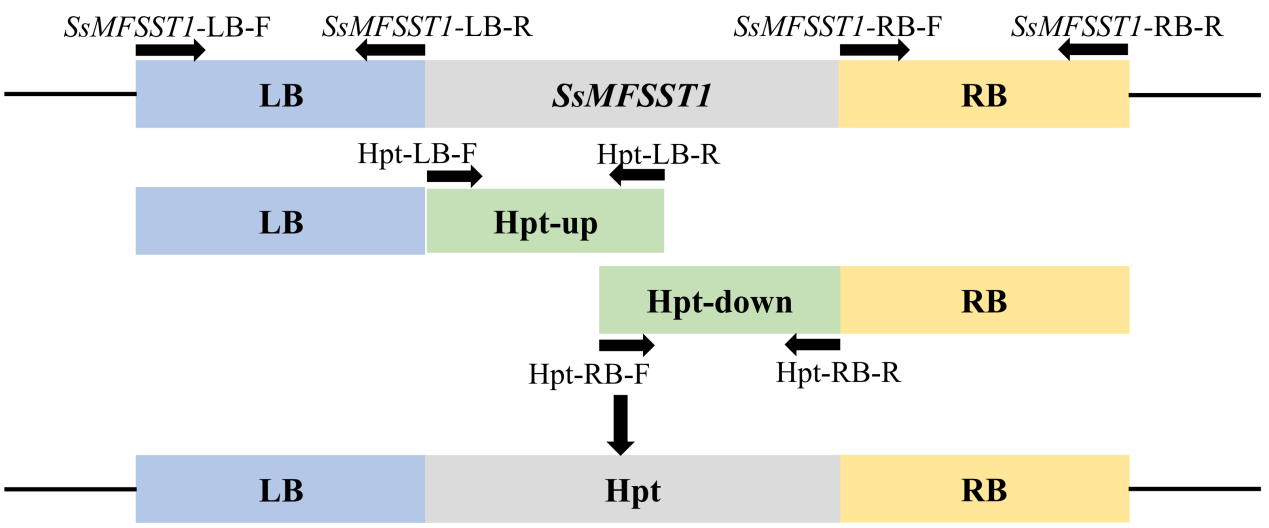
**

Fig. S1 Schematic diagram of gene knockout


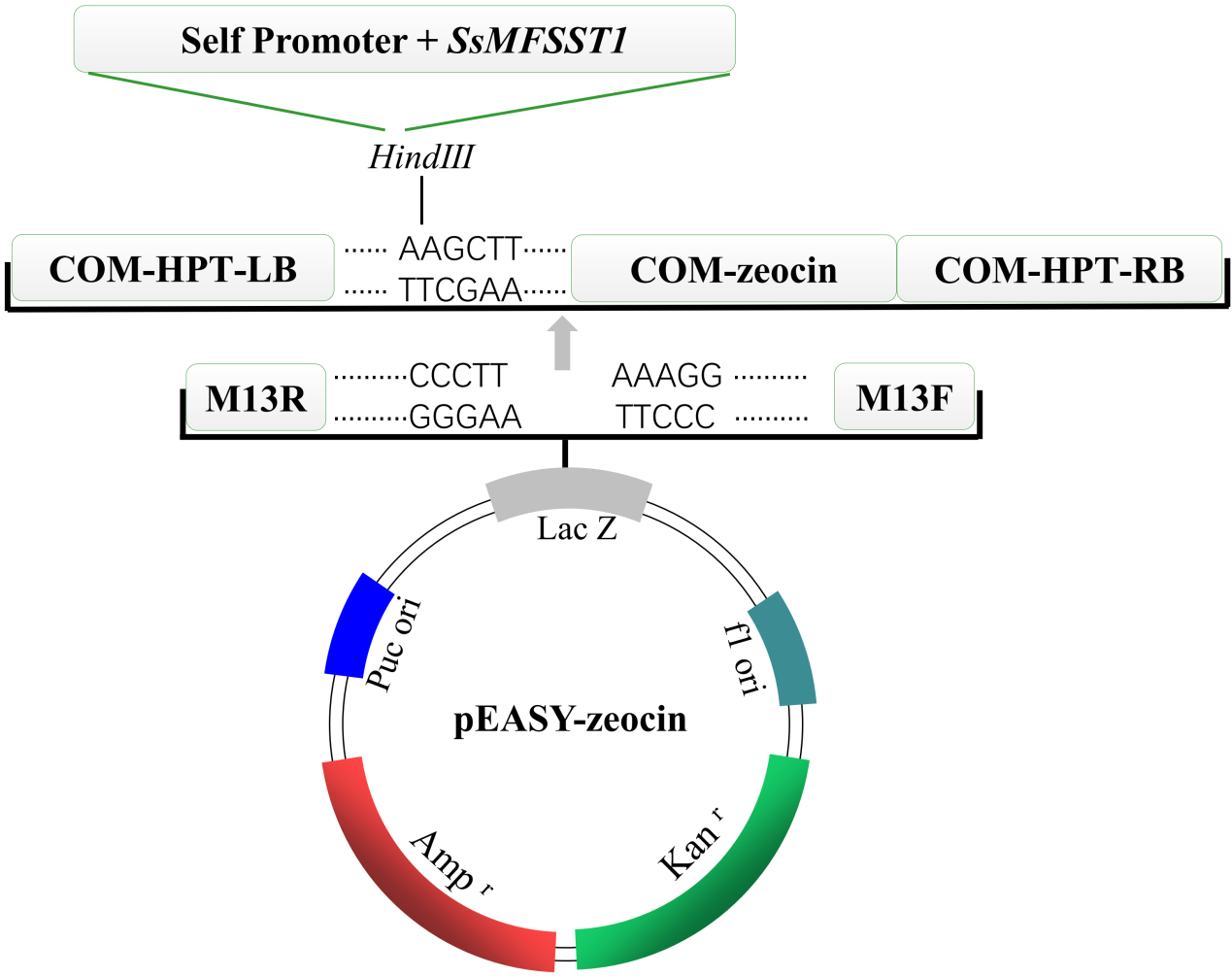


Fig. S2 Schematic diagram of gene complementation

**
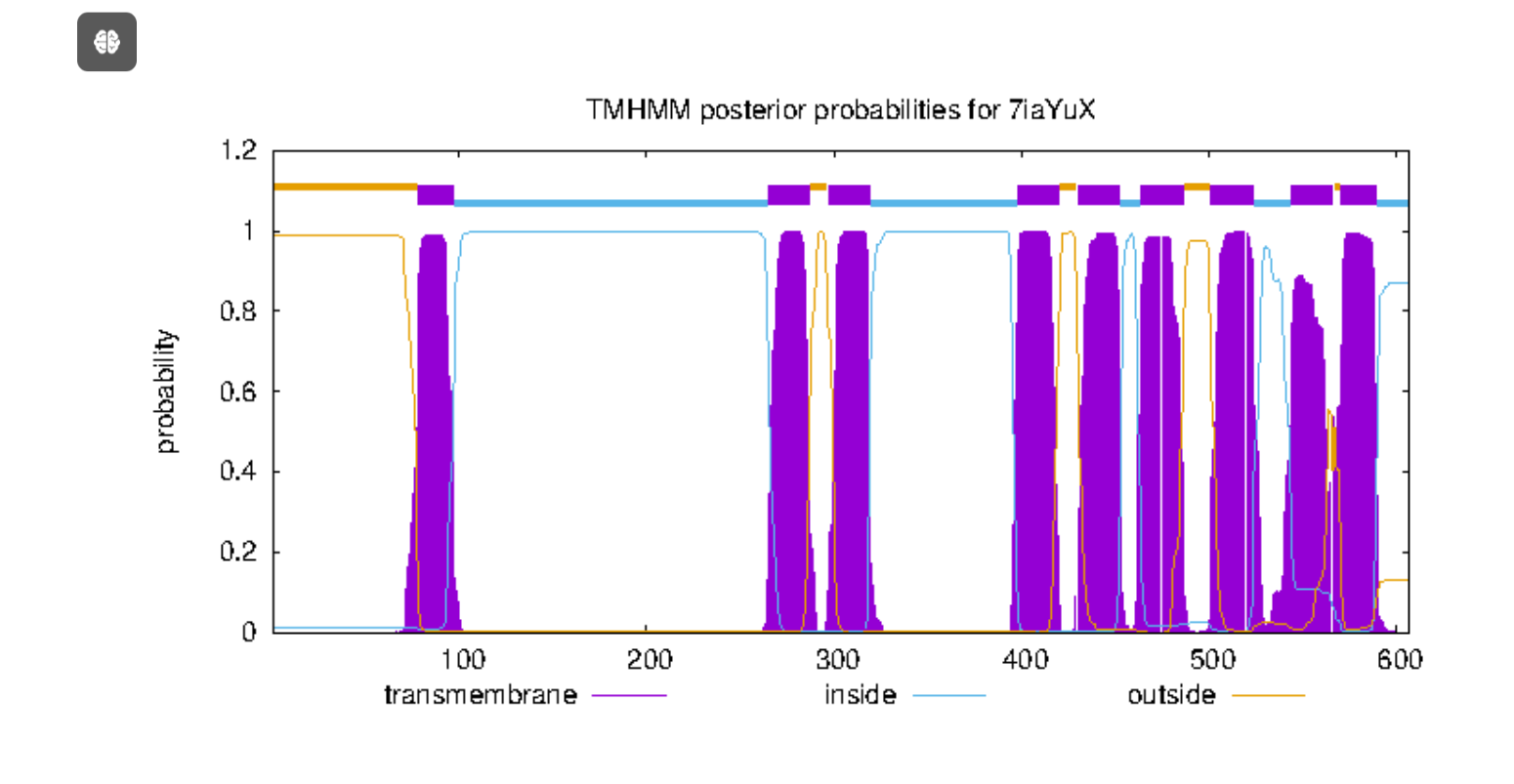
**

Fig. S3 The predicted transmembrane helices of SsMFSST1

**
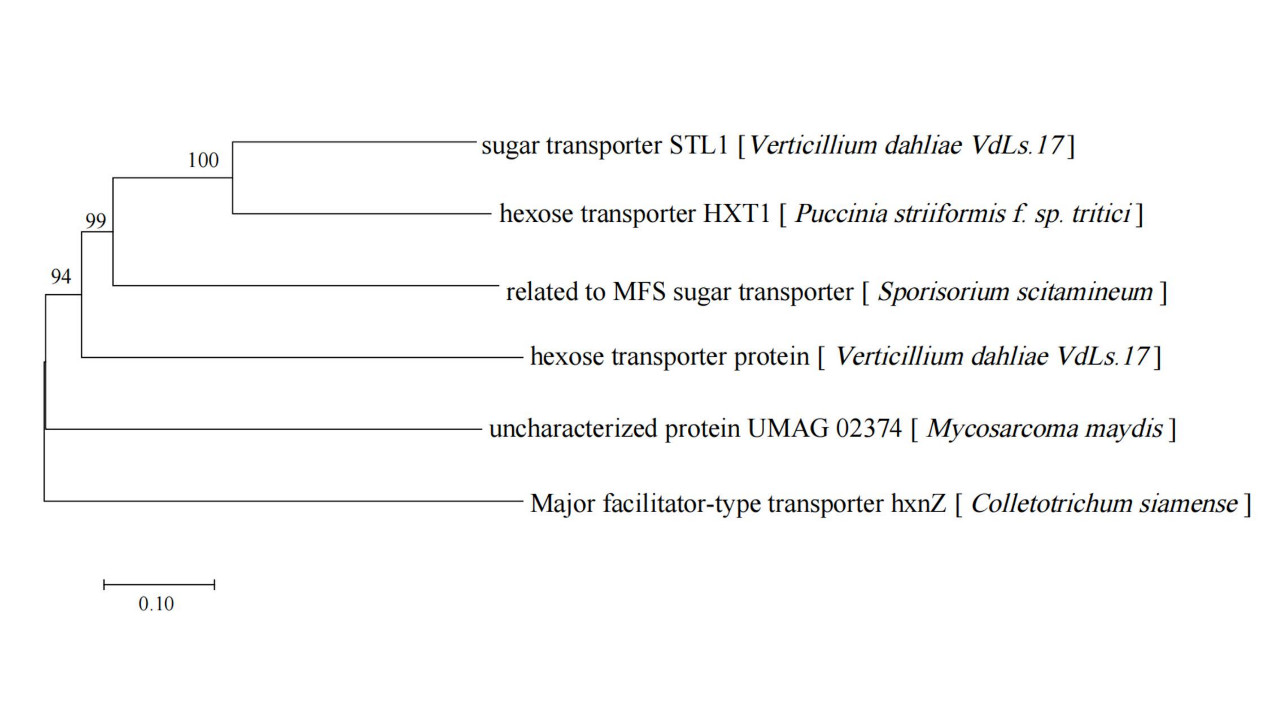
**

Fig.S4 The phylogenetic relationship between SsMFSST1 and MFS sugar transporters reported with role in pathogenesis in other fungi


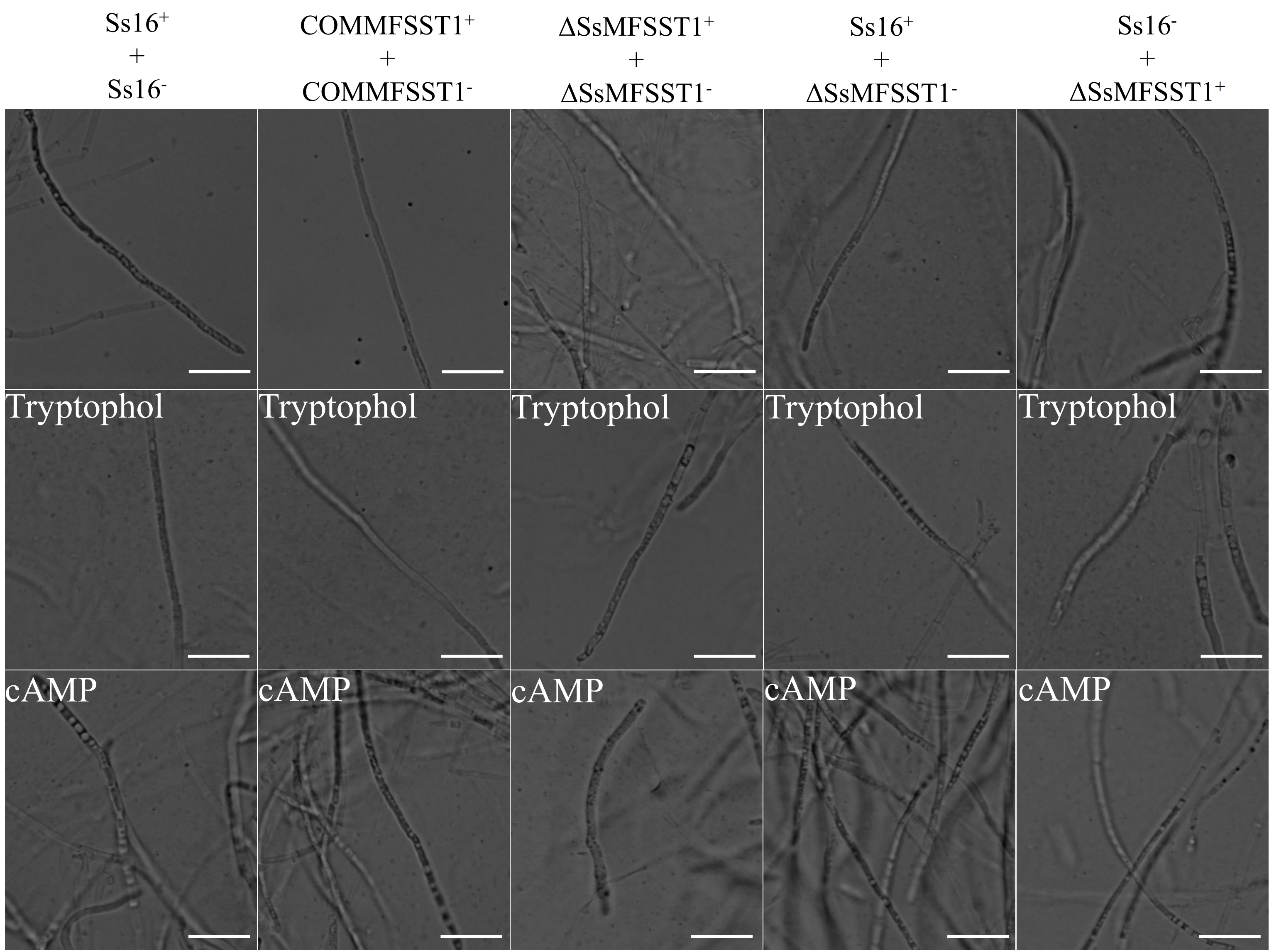


Fig. S5 Hyphae morphology under 40**×** microscope under different conditions. The white bars represent 25 μm.


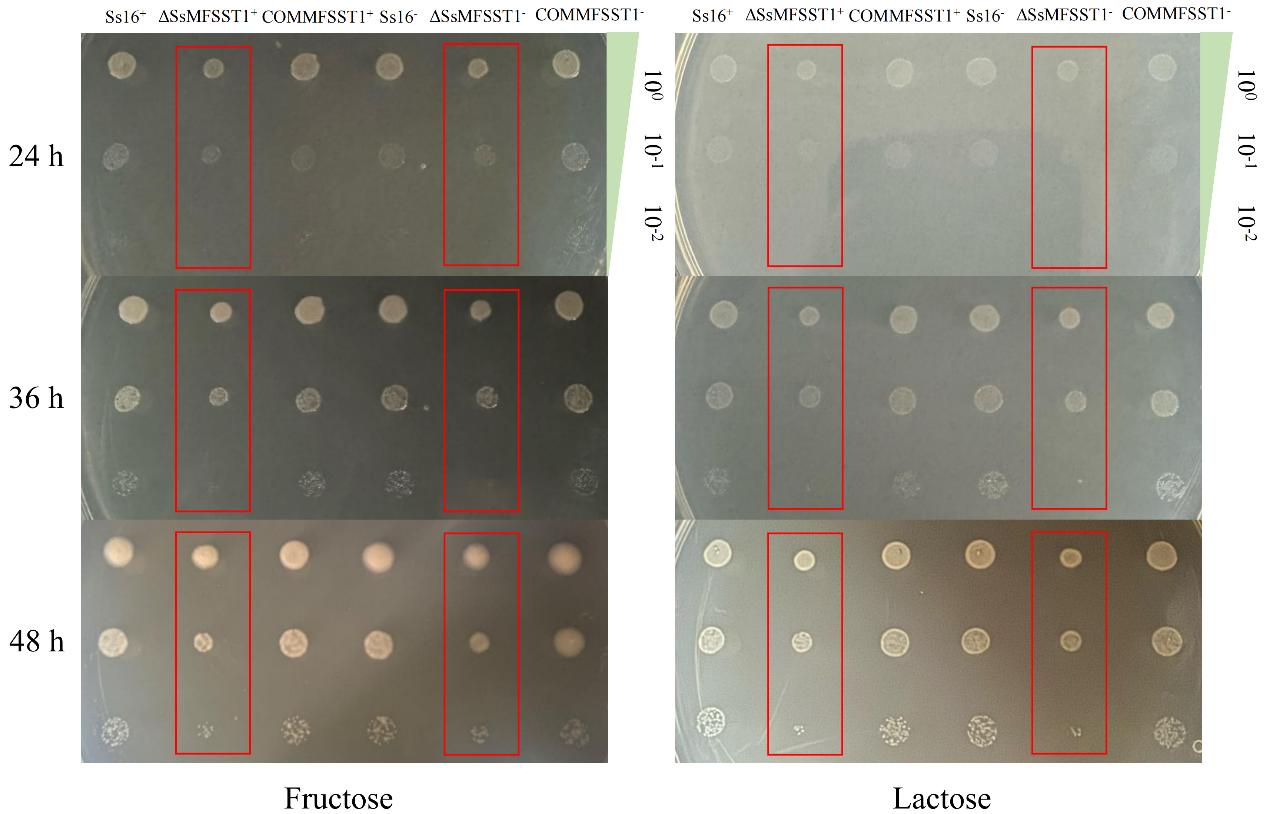


Fig. S6 The growth of colonies with fructose or lactose as the sole carbon source added to MM solid culture medium
